# Pseudo-pseudo Meigs Syndrome as the first clinical manifestation of systemic lupus erythematosus after controlled ovarian stimulation for ICSI: a case report

**DOI:** 10.3389/fimmu.2026.1936700

**Published:** 2026-10-22

**Authors:** Sicong Li, Dan Zhao, Mengpei Zhang

**Affiliations:** 1 Department of Emergency, West China Second University Hospital, Sichuan University, Chengdu, China; 2 Key Laboratory of Birth Defects and Related Diseases of Women and Children (Sichuan University), Ministry of Education, Chengdu, China; 3 Department of Physiology, West China School of Basic Medical Sciences and Forensic Medicine, Sichuan University, Chengdu, China; 4 Department of Cancer Center, West China Second University Hospital, Sichuan University, Chengdu, China

**Keywords:** assisted reproductive technology, ovarian hyperstimulation syndrome, ovarian malignancy, Pseudo-pseudo Meigs syndrome, systemic lupus erythematosus

## Abstract

**Background:**

Pseudo-pseudo Meigs syndrome (PPMS), or Tjalma syndrome, is a rare systemic lupus erythematosus (SLE)-associated phenotype characterized by ascites, pleural effusion, and elevated CA125 without an ovarian neoplasm. After assisted reproductive technology (ART), it may overlap with ovarian hyperstimulation syndrome (OHSS) or mimic gynecologic malignancy.

**Case presentation:**

A 25-year-old woman underwent two controlled ovarian stimulation (COS) cycles for intracytoplasmic sperm injection (ICSI) because of severe male-factor infertility. Before ART, she had autoimmune thyroid disease and transient urinary abnormalities, but no known SLE. During the second COS cycle, hypertension and albuminuria emerged. Following oocyte retrieval, she developed massive ascites, bilateral pleural effusions, adnexal abnormalities, and marked CA125 elevation, initially raising suspicion of OHSS and ovarian malignancy. Subsequent nephrotic-range proteinuria, profound hypoalbuminemia, hypocomplementemia, and SLE-associated autoantibodies supported active SLE with substantial renal involvement. Cytologic and microbiologic investigations provided no evidence of malignancy or infection. Following SLE-directed treatment, renal abnormalities and serosal effusions improved. At eight months, the adnexal abnormalities had completely resolved and CA125 had normalized, supporting a PPMS phenotype.

**Conclusion:**

Atypical or persistent post-ART effusions, particularly when accompanied by evolving renal or immunologic abnormalities, should prompt consideration of SLE-associated PPMS as an important differential diagnosis beyond OHSS and gynecologic malignancy. Longitudinal evaluation and multidisciplinary assessment may facilitate timely recognition of this rare phenotype and guide appropriate clinical management.

## 1 Introduction

Autoimmune diseases disproportionately affect women, particularly during the reproductive years (1). Systemic lupus erythematosus (SLE) is a prototypical systemic autoimmune disease with a strong female predominance and a broad spectrum of clinical manifestations, including serositis, nephritis, hematologic abnormalities, and reproductive complications (2). In women of reproductive age, disease activity, renal involvement, thrombosis risk, autoantibody status, and immunosuppressive treatment may also complicate fertility and pregnancy management, often requiring multidisciplinary care (3).

Assisted reproductive technology (ART), including *in vitro* fertilization (IVF) and intracytoplasmic sperm injection (ICSI), is an established treatment for infertility. Current reproductive health guidance supports the use of ART in selected women with rheumatic and musculoskeletal diseases, particularly when disease activity is quiescent and pregnancy-compatible treatment is in place (3). However, in routine infertility practice, autoimmune disease may be unrecognized when patients lack classic systemic symptoms at baseline. Controlled ovarian stimulation (COS), a central component of many ART protocols, also introduces a short period of marked endocrine and vascular changes. Although these changes do not establish causality for autoimmune disease onset, they may reveal previously subclinical immune or renal abnormalities in susceptible individuals (4, 5). Therefore, when unexpected systemic complications occur after ART, the diagnostic assessment should not be restricted to reproductive medicine complications alone.

Ovarian hyperstimulation syndrome (OHSS) remains the most familiar ART-related explanation for post-retrieval abdominal distension, ascites, ovarian enlargement, hypoalbuminemia, and pleural effusion (6). In parallel, marked cancer antigen 125 (CA125) elevation and adnexal or peritoneal abnormalities may raise concern for ovarian malignancy or Meigs-like syndromes. Pseudo-pseudo Meigs syndrome (PPMS), also known as Tjalma syndrome, is a rare manifestation of SLE characterized by ascites, pleural effusion, and elevated CA125 without an underlying ovarian tumor (7, 8). It may therefore mimic both gynecologic malignancy and ART-related complications, particularly when SLE has not been previously recognized (8, 9). Here, we report a young woman who developed SLE-associated PPMS after COS for ICSI (**Figure 1**). The case highlights PPMS as a potential post-ART diagnostic trap and the importance of integrating renal, serologic, and systemic clues when evaluating atypical post-ART effusions.

**Figure 1:**
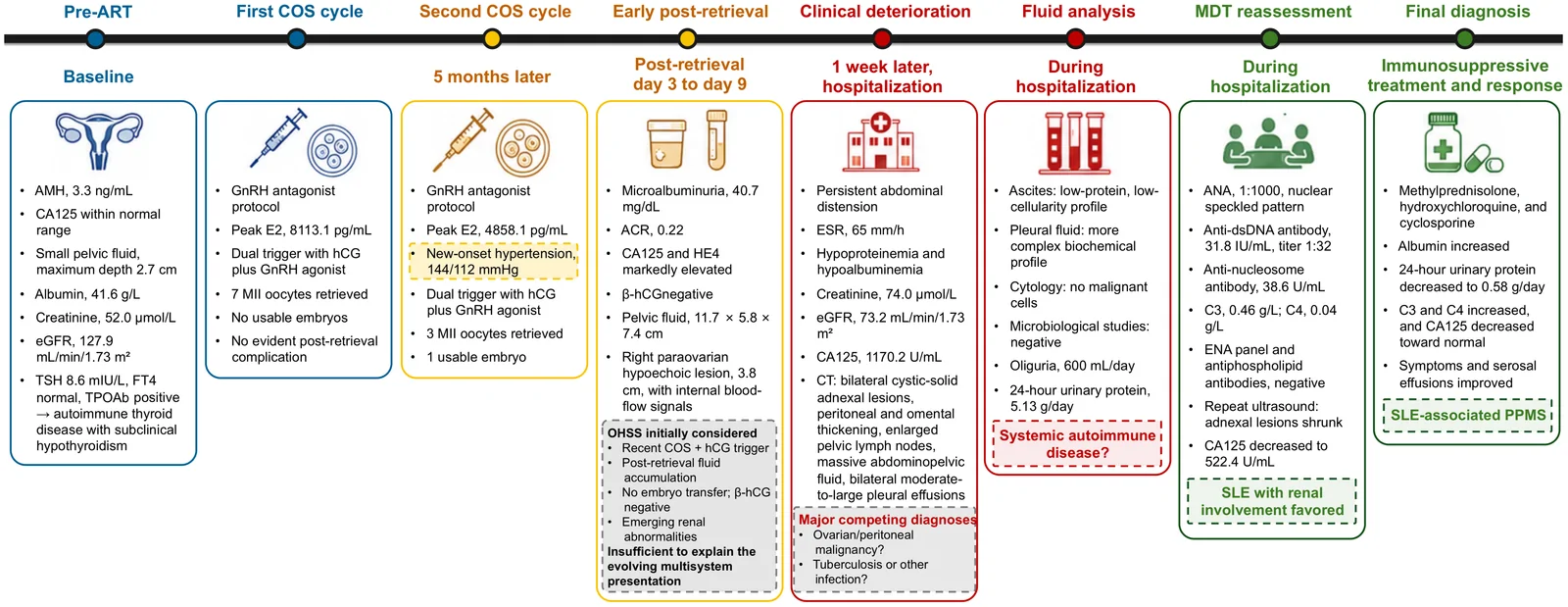
Clinical timeline and diagnostic evolution from assisted reproductive technology to systemic lupus erythematosus-associated pseudo-pseudo Meigs syndrome. The timeline summarizes the key clinical events, laboratory findings, imaging features, diagnostic pivots, and treatment response from pre-ART assessment to the final diagnosis. ACR, albumin-to-creatinine ratio; AMH, anti-Müllerian hormone; ANA, antinuclear antibody; anti-dsDNA, anti-double-stranded DNA; ART, assisted reproductive technology; CA125, cancer antigen 125; COS, controlled ovarian stimulation; CT, computed tomography; E2, estradiol; eGFR, estimated glomerular filtration rate; ENA, extractable nuclear antigen; ESR, erythrocyte sedimentation rate; FT4, free thyroxine; GnRH, gonadotropin-releasing hormone; hCG, human chorionic gonadotropin; HE4, human epididymis protein 4; ICSI, intracytoplasmic sperm injection; MDT, multidisciplinary team; MII, metaphase II; OHSS, ovarian hyperstimulation syndrome; PPMS, pseudo-pseudo Meigs syndrome; SLE, systemic lupus erythematosus; TPOAb, thyroid peroxidase antibody; TSH, thyroid-stimulating hormone.

## 2 Case presentation

A 25-year-old woman, gravida 1 para 0, with a history of one prior elective induced abortion, was referred to West China Second University Hospital, Sichuan University, for ICSI because of secondary infertility predominantly attributable to severe male-factor infertility. Her body mass index was 27.0 kg/m^2^. Her menstrual cycles were regular, and she reported no known history of reproductive endocrine disease, autoimmune disease, renal disease, malignancy, tuberculosis, or other chronic systemic illness. Baseline blood pressure was 110/68 mmHg.

### 2.1 Pre-ART assessment: reassuring findings with subtle systemic clues

The initial pre-stimulation assessment appeared largely reassuring. Ovarian reserve was preserved, with an anti-Müllerian hormone (AMH) level of 3.3 ng/mL, and transvaginal ultrasonography showed only a small amount of pelvic fluid, with a maximum depth of 2.7 cm. Serum CA125 was within the normal range. Baseline serum albumin, serum creatinine, and estimated glomerular filtration rate were 41.6 g/L, 52.0 μmol/L, and 127.9 mL/min/1.73 m^2^, respectively. Minor urinary sediment abnormalities, including erythrocytes and casts, resolved on repeat testing the following day and were attributed to menstrual contamination. Thyroid testing showed an elevated thyroid-stimulating hormone (TSH) level of 8.6 mIU/L, a normal free thyroxine (FT4) level of 14.4 pmol/L, and positive anti-thyroid peroxidase antibody (TPOAb), consistent with autoimmune thyroid disease presenting as subclinical hypothyroidism. Levothyroxine therapy was initiated, and thyroid function was normalized before COS. This was considered an isolated organ-specific autoimmune disorder at the time.

### 2.2 COS and early post-retrieval warning signs

After levothyroxine treatment and normalization of thyroid function, COS was initiated the following month using a gonadotropin-releasing hormone (GnRH) antagonist protocol. During the first stimulation cycle, serum estradiol peaked at 8113.1 pg/mL, and final oocyte maturation was induced with a dual human chorionic gonadotropin (hCG)/GnRH agonist trigger. Seven mature metaphase II (MII) oocytes were retrieved, but no usable embryos developed. No post-retrieval complications occurred.

Five months later, she underwent a second stimulation cycle using the same antagonist strategy. Thyroid function remained normal under levothyroxine treatment. During stimulation, serum estradiol increased to 4858.1 pg/mL. Near the end of stimulation, new-onset hypertension developed (144/112 mmHg) and was controlled with oral labetalol. After the same dual-trigger strategy, three MII oocytes were retrieved, and one usable embryo was obtained.

Because of the newly detected hypertension, an additional urinary assessment was performed and showed microalbuminuria (40.7 mg/dL; albumin-to-creatinine ratio [ACR], 0.22). Nine days after oocyte retrieval, CA125 and HE4 were markedly elevated, whereas β-hCG remained negative. Transvaginal ultrasonography showed substantial pelvic fluid, measuring 11.7 × 5.8 × 7.4 cm, and a right 3.8 cm paraovarian hypoechoic lesion with an ill-defined margin and internal blood-flow signals. No embryo transfer was performed.

Given the recent ovarian stimulation, hCG-containing trigger, and post-retrieval pelvic fluid accumulation, OHSS was initially considered. However, new-onset hypertension and microalbuminuria together with marked CA125 and HE4 elevation, and the paraovarian abnormality raised concern that OHSS alone might not explain the evolving presentation and broadened the differential diagnosis. A planned admission was arranged one week later for further evaluation.

### 2.3 Clinical deterioration and broadening differential diagnosis

One week later, she returned with persistent abdominal distension. Laboratory testing showed an elevated erythrocyte sedimentation rate (65 mm/h), normal white blood cell and platelet counts with mild anemia (hemoglobin, 102.0 g/L), profound hypoproteinemia and hypoalbuminemia, and worsening renal indices, with serum creatinine increasing to 74.0 μmol/L and estimated glomerular filtration rate (eGFR) declining to 73.2 mL/min/1.73 m^2^. CA125 increased further to 1170.2 U/mL, whereas β-hCG remained negative. Ultrasound showed persistent pelvic fluid, the previously noted right-sided adnexal lesion, and bilateral pleural effusions (**Figures 2A–C**). These findings raised concern for ovarian malignancy and broadened the differential diagnosis beyond OHSS. Tuberculosis was also considered because of the extensive serosal effusions and marked CA125 elevation.

**Figure 2:**
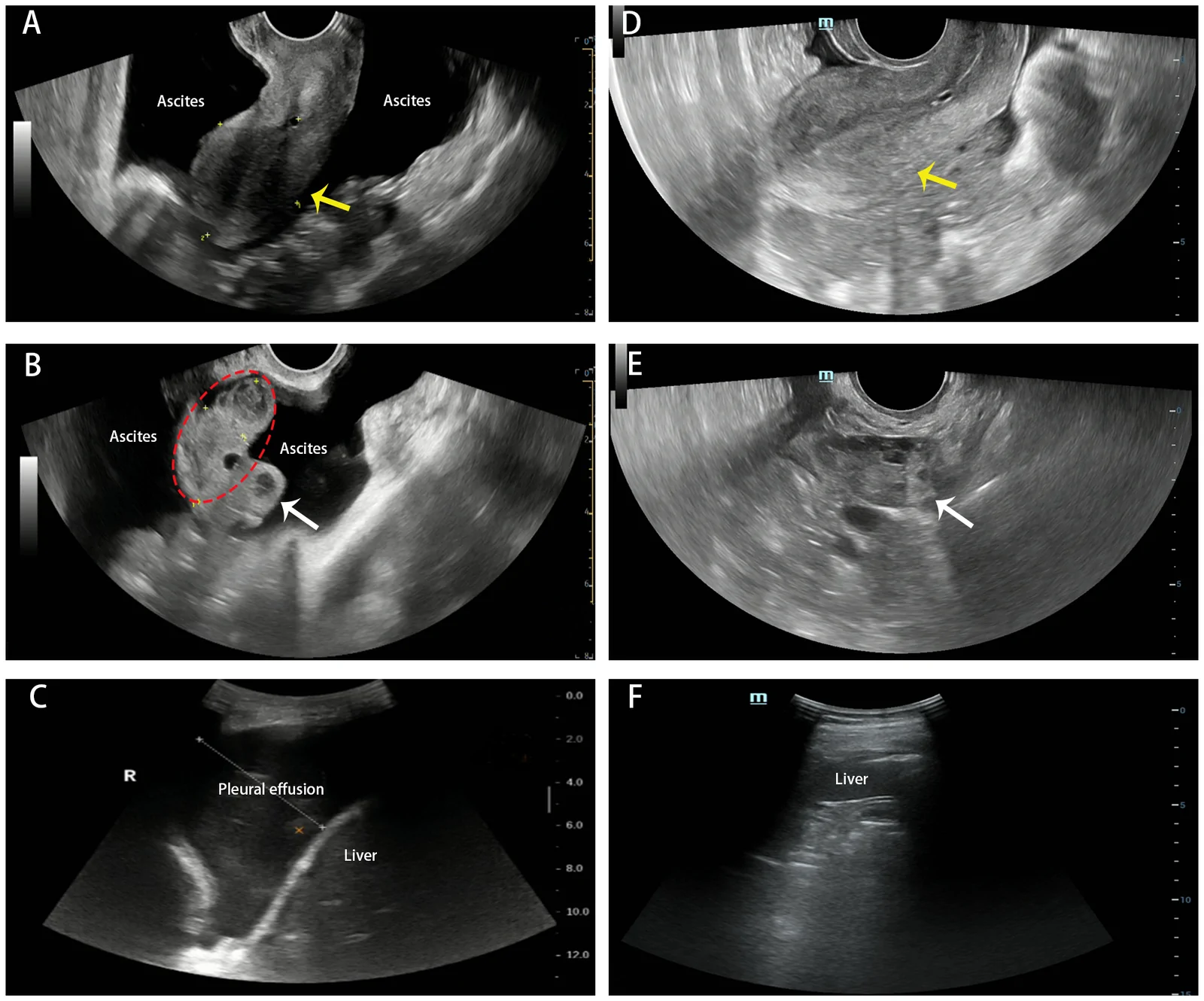
Longitudinal ultrasonographic resolution of pelvic and pleural abnormalities. **(A–C)** Ultrasonographic findings during the acute phase before SLE-directed treatment. **(A)** Transvaginal ultrasonography showing extensive pelvic ascites surrounding the uterus. **(B)** Transvaginal ultrasonography showing persistent ascites and a right paraovarian lesion (red dashed circle). **(C)** Thoracic ultrasonography showing a substantial pleural effusion. **(D–F)** Ultrasonographic findings at approximately 8 months after SLE-directed treatment. **(D)** Transvaginal ultrasonography showing resolution of pelvic ascites. **(E)** Transvaginal ultrasonography showing complete resolution of the previously observed paraovarian lesion. **(F)** Thoracic ultrasonography showing resolution of the pleural effusion. Yellow arrows indicate the uterus, and white arrows indicate the ovaries. SLE, systemic lupus erythematosus.

Supportive treatment was started, including albumin supplementation, diuretics. Diagnostic paracentesis showed low-protein, low-cellularity ascites with a negative Rivalta test. Cytologic and microbiologic examinations showed no evidence of malignancy or infection, including tuberculosis. Thoracentesis was subsequently performed for symptomatic pleural effusion. The pleural-to-serum total protein ratio was 0.514, meeting the protein-ratio component of Light’s criteria for an exudate, whereas the absolute pleural-fluid protein concentration (20.2 g/L), lactate dehydrogenase level (68.0 U/L), and nucleated cell count (83 ×10^6^/L) were relatively low. Pleural-fluid cytology and microbiologic testing were also negative.

Despite supportive care, hypoalbuminemia persisted and proteinuria progressed to 5.13 g/day, reaching the nephrotic range. Together with the low-protein ascitic fluid and the profound hypoalbuminemia, these findings raised increasing concern for a renal and systemic process rather than an isolated post-retrieval complication.

### 2.4 Diagnostic pivot: renal protein loss and systemic autoimmunity

A multidisciplinary discussion was convened to reassess the diagnosis. Although a limited early contribution from OHSS could not be excluded, persistent β-hCG negativity without embryo transfer, together with progressive renal abnormalities and extensive serosal effusions, made isolated OHSS increasingly insufficient as the principal explanation. Exploratory surgery was discussed because of the adnexal findings and markedly elevated CA125. However, severe hypoalbuminemia and massive effusions increased the procedural risk, the immediate surgery was deferred. Repeat transvaginal ultrasonography showed regression of the adnexal abnormalities, while CA125 transiently decreased to 522.4 U/mL. These dynamic changes further weakened the likelihood of ovarian malignancy.

The evolving renal and systemic findings prompted autoimmune evaluation. Testing showed antinuclear antibody (ANA) 1:1000 with a nuclear speckled pattern, positive anti-double-stranded DNA (dsDNA) antibody (31.8 IU/mL, titer 1:32), positive anti-nucleosome antibody (38.6 U/mL), low complement levels (C3, 0.46 g/L; C4, 0.04 g/L). The remaining extractable nuclear antigen panel and antiphospholipid antibody testing were negative. The direct antiglobulin (Coombs) test was negative.

Renal biopsy was considered but could not be performed during the acute phase, because of the patient’s severe clinical condition, including massive serosal effusions and profound hypoalbuminemia. After clinical stabilization, the procedure was reconsidered, but the patient declined. Consequently, the histopathologic class and activity of the renal involvement remained undetermined.

Taken together, the clinical presentation and laboratory findings supported active SLE with substantial renal involvement (**Table 1**), presenting with massive ascites, pleural effusions, markedly elevated CA125, transient adnexal abnormalities, and no evidence of malignancy or infection, a constellation most consistent with a PPMS phenotype. Renal protein loss and hypoalbuminemia were considered important contributors to the effusions, although the differing pleural and ascitic fluid profiles suggested more than one mechanism.

**Table 1:** Clinical and immunologic features supporting SLE classification according to the 2019 EULAR/ACR criteria.

2019 EULAR/ACR criteria	Evidence in this patient	Points counted	Total score
Entry criterion: ANA ≥1:80 at least once	Yes, ANA 1:1000		
Clinical domain			19 (≥10 threshold met)
Constitutional	No	0	
Hematologic	No	0	
Neuropsychiatric	No	0	
Mucocutaneous	No	0	
Serosal	Yes, pleural effusion	5	
Musculoskeletal	No	0	
Renal	Yes, proteinuria 5.13 g/day	4	
Immunology domain			
Antiphospholipid antibodies	No	0	
Complement proteins	Yes, low C3 (0.46 g/L) and low C4 (0.04 g/L)	4	
SLE-specific antibodies	Yes, positive anti-dsDNA antibody	6	

ANA, antinuclear antibody; anti-dsDNA, anti-double-stranded DNA antibody; C3, complement component 3; C4, complement component 4; EULAR, European Alliance of Associations for Rheumatology; ACR, American College of Rheumatology; SLE, systemic lupus erythematosus.

### 2.5 Treatment response and confirmation of SLE-associated PPMS

Given the substantial renal involvement, nephrotic-range proteinuria, and the patient’s ongoing reproductive goals, the treating team selected cyclosporine as part of an individualized regimen. Because renal histology was unavailable, treatment was guided by the clinical renal phenotype rather than a biopsy-defined lupus nephritis class. SLE-directed therapy was initiated using intravenous methylprednisolone pulses (500 mg once daily for three consecutive days), hydroxychloroquine (200 mg twice daily), and cyclosporine (2.5 mg/kg/day, 50 mg in the morning and 75 mg in the evening). After clinical stabilization, methylprednisolone was transitioned to an oral dose of 40 mg daily. Supportive treatment included albumin infusion, diuretics, antihypertensive therapy, and thromboprophylaxis as clinically indicated.

After two weeks, abdominal distension and pleural symptoms improved substantially. Serum albumin increased from 13.8 g/L to 25.6 g/L, 24-hour urinary protein decreased from 5.13 g/day to 0.58 g/day, serum creatinine was 65 μmol/L, and complement C3 and C4 increased toward the normal range. Serosal effusions and CA125 also decreased. At the one-month follow-up, only minimal residual ascites remained, bilateral ovarian morphology had returned to normal, and CA125 had decreased to approximately the upper limit of normal (ULN).

At approximately eight months after discharge, the patient remained clinically stable on hydroxychloroquine 200 mg/day, cyclosporine 2.0 mg/kg/day (50 mg twice daily), and methylprednisolone 4 mg/day, without further COS or embryo transfer. Follow-up ultrasound showed complete resolution of the previously observed adnexal lesions, ascites, and pleural effusions (**Figure 2D–F**), and serum CA125 was 20.0 U/mL. Serum albumin was 35.0 g/L, 24-hour urinary protein of 0.09 g/day, serum creatinine of 62.0 μmol/L, and an eGFR of 106.0 mL/min/1.73 m^2^. C3 and C4 increased to 0.79 and 0.20 g/L, respectively, and anti-dsDNA antibody became negative.

The sustained resolution of the adnexal abnormalities and serosal effusions, normalization of CA125, and parallel renal and immunologic improvement further argued against ovarian malignancy and supported the diagnosis of active SLE with substantial renal involvement presenting with a PPMS phenotype.

## 3 Discussion

This case illustrates an uncommon but clinically important diagnostic challenge in the post-ART setting. Following COS, the combination of serosal effusions, adnexal abnormalities, and marked CA125 elevation initially suggested OHSS or ovarian malignancy. However, progressive renal involvement, hypocomplementemia, and SLE-associated serologic abnormalities, together with the absence of evidence for malignancy or infection, indicated a broader systemic process. The overall presentation was most consistent with active SLE with renal involvement, manifesting as a PPMS phenotype.

OHSS was a plausible initial diagnosis because ascites and pleural effusions developed after oocyte retrieval following an hCG-containing trigger, consistent with the increased vascular permeability characteristic of OHSS (10, 11). However, new-onset hypertension, progressive nephrotic-range proteinuria, hypocomplementemia, and SLE-associated serologic abnormalities increasingly favored an underlying systemic autoimmune process over isolated OHSS. The absence of embryo transfer and persistently negative β-hCG further argued against pregnancy-associated late-onset OHSS (12). Thus, a limited early contribution from OHSS could not be excluded, but OHSS alone was insufficient to explain the evolving multisystem presentation.

Marked CA125 elevation, extensive serosal effusions, adnexal abnormalities, and peritoneal changes raised concern for ovarian or peritoneal malignancy. Peritoneal or genital tuberculosis was another important consideration because it can produce similar clinical and imaging findings (13–16). However, CA125 is not tumor-specific and may increase with mesothelial activation, serosal inflammation, and fluid accumulation (17–19). In this patient, fluid cytology revealed no malignant cells, and targeted microbiological investigations provided no evidence of tuberculosis or other infection. Furthermore, serial imaging demonstrated progressive regression and eventual resolution of the adnexal abnormalities, accompanied by normalization of CA125 after SLE-directed treatment, further reduced the likelihood of an underlying malignancy.

In the context of active SLE, the combination of ascites, pleural effusions, and marked CA125 elevation, without evidence of an ovarian neoplasm, supported a PPMS (Tjalma syndrome) phenotype (8, 20). Importantly, PPMS represents a clinical phenotype rather than as a condition defined by a single mechanism of fluid accumulation. Renal involvement has also been documented in patients with PPMS. Among 33 cases reviewed by Li and Tian, impaired renal function, hypoproteinemia, and proteinuria were reported in 39%, 58%, and 30%, respectively (9). Another report described PPMS associated with biopsy-confirmed class V lupus nephritis and nephrotic-range proteinuria (21). In our patient, nephrotic-range proteinuria, profound hypoalbuminemia, and low-protein ascites suggested a substantial contribution from renal protein loss and reduced plasma oncotic pressure. However, the differing biochemical profiles of the pleural and ascitic fluids left open the possibility of additional contributions from SLE-related serosal inflammation and altered vascular permeability (9, 22). Thus, the PPMS phenotype reflected the overall clinical presentation, while renal protein loss and possible serosal involvement provided complementary explanations for the effusions.

The distinctive feature of this case is the post-ART diagnostic context rather than the PPMS phenotype itself. Previously reported PPMS cases have largely involved presentations mimicking gynecologic malignancy, tuberculosis, or acute surgical disease (**Table 2**) (9, 23, 24). To our knowledge, PPMS has not previously been reported in close temporal association with COS. In the present patient, recent hCG-containing stimulation and subsequent fluid accumulation made OHSS a clinically plausible initial diagnosis, while marked CA125 elevation and adnexal abnormalities raised concern for ovarian malignancy. This dual diagnostic challenge was further complicated by the absence of a recognized SLE diagnosis before ART. The case illustrates how an expected reproductive complication may obscure the recognition of underlying systemic disease.

**Table 2:** Comparison of clinical context and diagnostic features between previously reported PPMS cases and the present case.

Characteristic	Previously reported PPMS cases (8, 9, 20–22, 24, 30–54) (n = 34)	Present case
Demographic and autoimmune context		
Age, years	14-82	25
Female sex	34/34 (100%)	Female
Established SLE before PPMS	12/34 (35.3%)	No
PPMS leading to first SLE diagnosis	22/34 (64.7%)	Yes
Other autoimmune/rheumatic disease before PPMS	9/34 (26.5%)	Autoimmune thyroid disease
Reproductive/hormonal context		
Pregnancy/postpartum reported	1/34 (2.9%)	No
ART/COS reported	0/34 (0%)	Yes, two COS cycles for ICSI
Major diagnostic challenge		
Ovarian/gynecologic malignancy considered	34/34 (100%)	Yes
Tuberculosis/other infection considered	22/34 (64.7%)	Yes
Acute surgical abdomen/other surgical disease considered	2/34 (5.9%)	No
OHSS considered	0/34 (0%)	Yes
Renal involvement		
Any reported renal involvement*	19/34 (55.9%)	Yes
Proteinuria	16/34 (47.1%)	Yes
Nephrotic-range proteinuria/nephrotic syndrome	9/34 (26.5%)	Yes
Biopsy-confirmed lupus nephritis	3/34 (8.8%)	No biopsy
Pelvic/adnexal findings		
Pelvic/adnexal abnormality reported	8/34 (23.5%)	Yes, transient

Values are n/34 (%) unless otherwise indicated. For literature-derived characteristics, n denotes the number of cases in which the feature was explicitly reported among the 34 published cases included in this comparison; unreported information was not interpreted as confirmed absence. Any reported renal involvement (*) was defined as at least one reported renal feature among elevated creatinine, proteinuria/nephrotic-range proteinuria, or biopsy-confirmed lupus nephritis. PPMS, pseudo-pseudo Meigs syndrome; SLE, systemic lupus erythematosus; ART, assisted reproductive technology; COS, controlled ovarian stimulation; ICSI, intracytoplasmic sperm injection; OHSS, ovarian hyperstimulation syndrome.

The temporal relationship between COS and the subsequent diagnosis of SLE requires careful interpretation. Autoimmune thyroid disease and transient urinary abnormalities before ART raised the possibility of pre-existing immune or renal abnormalities, although neither established subclinical SLE. SLE-associated autoantibodies may precede clinical diagnosis by several years (25), supporting the plausibility of previously unrecognized autoimmune disease. In patients with known SLE or antiphospholipid syndrome, ART-related risks of disease flare and thrombosis warrant particular attention, particularly when disease activity is not well controlled (3, 4). Against this background, the present case raises a hypothesis-generating question as to whether the rapid endocrine changes accompanying COS might interact with pre-existing immune dysregulation and facilitate the clinical unveiling of otherwise subclinical autoimmune disease. However, an interaction cannot be established from a single case. Rather than implicating COS as a causal trigger, we interpret the temporal association as potentially unmasking an underlying autoimmune process. This distinction is particularly relevant because the reproductive years overlap with the period in which SLE and other systemic autoimmune diseases commonly become clinically apparent (26, 27).

Rather than suggesting routine autoimmune screening for all ART candidates, this case highlights the need for targeted systemic reassessment when the post-retrieval course is atypical. Persistent or otherwise unexplained effusions, particularly when accompanied by new renal abnormalities, profound hypoalbuminemia, or hypocomplementemia, should prompt consideration of SLE and other systemic causes beyond OHSS and gynecologic malignancy. Infectious mimics, including tuberculosis when clinically relevant, should also be evaluated before immunosuppression. The value of multidisciplinary assessment was particularly evident in this case. OHSS, ovarian malignancy, infection, and active SLE were not merely diagnostic alternatives; each implied a substantially different therapeutic pathway. Integration of reproductive, oncologic, renal, rheumatologic, radiologic, and infectious-disease findings allowed these competing explanations to be progressively reweighted as the clinical course evolved.

Treatment selection in this patient also warrants consideration of renal disease severity and future reproductive plans. Cyclosporine was selected in the setting of nephrotic-range proteinuria and is considered compatible with pregnancy, although its potential nephrotoxic and hypertensive effects require monitoring (28). Azathioprine is likewise pregnancy-compatible but is generally used for maintenance rather than initial treatment of active proliferative lupus nephritis (28). Current EULAR recommendations favor glucocorticoids combined with mycophenolate or low-dose intravenous cyclophosphamide for active proliferative lupus nephritis, with the B-cell-targeted agent belimumab or a calcineurin inhibitor, particularly voclosporin or tacrolimus, considered as add-on therapy (29). In our patient, the absence of renal histology precluded treatment selection according to lupus nephritis class. Had the response been inadequate, renal biopsy would have been reconsidered, subject to clinical feasibility and patient consent, to guide escalation toward a mycophenolate- or cyclophosphamide-based regimen, potentially incorporating belimumab or an alternative calcineurin inhibitor.

This report has limitations. As a single case without pre-ART autoimmune testing, it cannot determine whether subclinical SLE preceded COS or establish a causal relationship between COS and the subsequent clinical presentation. Renal biopsy could not be performed during the acute phase because of the patient’s severe clinical condition and was subsequently declined after stabilization, leaving the histopathologic class and activity or chronicity of renal involvement undetermined and precluding biopsy-guided treatment. Finally, the adnexal abnormalities were not histologically evaluated because surgery was not performed. Nevertheless, their complete resolution on follow-up imaging, normalization of CA125, and negative fluid cytology provided longitudinal evidence against an underlying malignancy.

## 4 Conclusion

This case highlights SLE-associated PPMS as a rare but clinically important diagnostic challenge after ART, where it may overlap clinically with OHSS and mimic ovarian malignancy. OHSS may contribute to early post-retrieval fluid accumulation, atypical or persistent effusions accompanied by evolving renal or immunologic abnormalities should prompt evaluation for underlying systemic disease rather than attribution to OHSS alone. COS-related physiological changes may have coincided with or contributed to the clinical emergence of previously unrecognized SLE, although causality cannot be established. Longitudinal evaluation and multidisciplinary assessment may facilitate timely recognition of PPMS and appropriate clinical management.

## Data Availability

The raw data supporting the conclusions of this article will be made available by the authors, without undue reservation.
